# Identification of an appropriate reference gene for normalization of qRT-PCR expression analyses in human breast cancer cell lines: application to L-arginine depletion studies

**DOI:** 10.1007/s00432-025-06165-2

**Published:** 2025-03-26

**Authors:** Antonia Röglin, Rainer Böger, Fiona Kleinsang, Juliane Hannemann

**Affiliations:** Institute of Clinical Pharmacology and Toxicology, University Medical Center Hamburg-Eppendorf, Hamburg, Germany

**Keywords:** Breast cancer, Quantitative real-time PCR (qRT-PCR), Reference gene, Normalization, Gene expression analysis, L-arginine depletion

## Abstract

**Purpose:**

Quantitative real-time PCR (qRT-PCR) represents a robust methodology to investigate alterations in gene expression patterns during tumorigenesis. The quantification of target gene expression is conventionally standardized through normalization against a stably expressed reference gene. However, the expression profile of a specific reference gene can exhibit variability across different tissue types and diverse physiological conditions. This study aimed to identify a suitable reference gene from a pool of ten potential candidates for the comparison of gene expression profiles between six human breast cell lines, comprising both normal breast (MCF-12A) and breast cancer cells (MCF-7, BT-474, SK-BR-3, MDA-MB-468, MDA-MB-231).

**Methods:**

Four different mathematical approaches were used to calculate the stability of reference gene expression (comparative ΔCt method, NormFinder, coefficient of variation and RefFinder).

**Results:**

Stability analysis identified *ACTB* as a suitable reference gene across all cell lines. As we are specifically interested in studying metabolic adaptation of breast cancer, we applied the same approach to identify a suitable reference gene also after maintaining the cell lines in L-arginine-deficient medium for up to 72 h. The stability ranking of reference genes fluctuated after L-arginine was depleted.

**Conclusion:**

In the context of investigating specific cell lines under certain conditions, we propose the identification of reference genes that exhibit optimal stability and suitability.

## Introduction

Due to its reproducibility, sensitivity, high throughput, and ease of use, quantitative real-time polymerase chain reaction (qRT-PCR) is an effective method for detecting differences in gene expression or determining the abundance of transcripts (Bustin 2000; Ginzinger 2002). This assay normalizes gene expression using an internal reference gene. An appropriate reference gene should be adequately abundant and exhibit stable expression across various tissues and cell lines under the chosen experimental conditions (Vandesompele et al. 2002). Inadequate quantification and incorrect conclusions can result from inaccurate normalization (Rho et al. 2010; Vandesompele et al. 2002). Several analyses, such as the comparative ΔCt method (Silver et al. 2006), NormFinder (Andersen et al. 2004), geNorm (Vandesompele et al. 2002), coefficient of variation (Boda et al. 2009) and RefFinder, have been proposed to determine appropriate reference genes in previous studies. With respect to breast cancer, identification of valid reference genes for normalization has been performed in several studies by comparing normal tissues and breast tumor tissues ex vivo (De Kok et al. 2005; Gur-Dedeoglu et al. 2009; Kiliç et al. 2014; Lyng et al. 2008; Majidzadeh-A et al. 2011; McNeill et al. 2007), but only few studies have evaluated reference genes for representative cell lines in vitro (Liu et al. 2015; Lyng et al. 2008; Morse et al. 2005). However, gene expression profiles in stable cancer cell lines differ in many aspects from those in tumor tissues ex vivo (Sandberg and Ernberg 2005). Similar differences in gene expression profiles exist between different tumors and between cell lines representing different tumors. Accordingly, it is essential for an appropriate reference gene to have stable expression across the studied tissues or cell lines under the specific experimental conditions (Vandesompele et al. 2002).

Breast cancer is recognized to be a group of diseases rather than a single, homogeneous disease (Cappelletti et al. 2017). To date, four molecular subtypes are commonly differentiated by morphological, molecular and clinical heterogeneity and can be distinguished by microarray techniques and gene expression studies (Perou et al. 2000; Sørlie et al. 2003): luminal A, luminal B, HER2-enriched and basal-like breast cancer. Clinically, this heterogeneity is mirrored by differences in relapse-free and overall survival (Sørlie et al. 2001, 2003). In order to enable gene expression studies across breast cancer cell lines representing these heterogeneous biological subtypes of the disease, we performed a screening study to identify a suitable reference gene that is stably expressed across all six cell lines that we included in our study.

Metabolic adaptation is recognized as one of the hallmarks of cancer; it has recently attracted attention as a means to sensitize cancer cells against therapeutic agents (Pavlova et al. 2022; Xia et al. 2021). L-arginine is a semi-essential amino acid that is involved in protein metabolism, cell proliferation, and that acts as a substrate for arginases (ARG) and nitric oxide synthases (NOS) (Böger 2007; Morris 2007). As L-arginine supply has been shown to become essential in rapidly growing tissues and organisms (Wu et al. 2009), L-arginine depletion has been tested in some tumors as a therapeutic intervention (Chu et al. 2023). However, previous studies on the influence of L-arginine on tumor growth in breast cancer resulted in contradictory findings (Brittenden et al. 1994; Cao et al. 2016; Garlick & McNurlan 1994; Park et al. 1992). This may relate to the fact that the intrinsic subtypes of breast cancer were often not appropriately considered. We therefore set out to identify the most stably expressed reference gene for normalization in six human breast cell lines in the presence or absence of the semi-essential amino acid, L-arginine.

## Material and methods

### Cell lines and culture conditions

Breast cells were purchased from American Type Culture Collection (ATCC, Manassas, Virginia, USA). MCF-7, BT-474, SK-BR-3, MDA-MB-468 and MDA-MB-231 cells were cultured in RPMI 1640 medium supplemented with 10% fetal bovine serum (FBS), 100 units/ml penicillin and 100 μg/ml streptomycin (Sigma-Aldrich, St. Louis, USA). The immortalized breast epithelial cell line MCF-12A was cultured in DMEM/F-12 medium supplemented with 5% horse serum (HS), 100 units/ml penicillin, 100 μg/ml streptomycin, 100 ng/ml cholera toxin (Merck KGaA, Darmstadt, Germany), 20 ng/ml human epidermal growth factor (hEGF, ThermoFisher Scientific, Waltham, MA, USA), 0.01 mg/ml insulin (Merck KGaA, Darmstadt, Germany), and 500 ng/ml hydrocortisone (STEMCELL Technologies Canada Inc., Vancouver, British Columbia, Canada). Cholera toxin, hEGF, insulin, and hydrocortisone were added immediately before use. All cells were cultured in a humidified environment with 5% CO_2_ saturation at 37 °C.

### RNA isolation and cDNA synthesis

Cells were harvested by cell scraping, transferred into 1 ml cold TRIzol™ (ThermoFisher Scientific, Waltham, MA USA), and stored at −80 °C until further use. Removal of genomic DNA (gDNA) and sample clean-up were carried out utilizing the PureLink™ RNA Mini Kit in combination with PureLinkTM DNase (Thermo Fisher Scientific) according to the manufacturer’s instructions. Extracted RNAs were quantified with an N60 NanoPhotometer (Implen, Munich, Germany) at a wavelength of 260 nm. RNA purity and integrity was verified by agarose gel electrophoresis. Total RNA (2.5 µg) was reverse-transcribed using the SuperScript™ IV VILO™ Master Mix (ThermoFischer) according to the manufacturer’s instructions. The reaction mix contained 4 µl of SuperScript MasterMix and 2.5 µg of template RNA, diluted in nuclease-free water to a volume of 14 µl.

### Selection of reference genes

Table 1 gives an overview of the potential reference genes selected for this study and their respective molecular function. *ACTB, PPIA, PUM1, SDHA* and *TBP* genes were selected as candidate reference genes because they had been used in studies comparing breast cancer and normal breast tissue ex vivo (De Kok et al. 2005; Gur-Dedeoglu et al. 2009; Kiliç et al. 2014; Lyng et al. 2008; Majidzadeh-A et al. 2011; McNeill et al. 2007; Morse et al. 2005; Salimi et al. 2012). Furthermore, *GAPDH* was included into the study because it had been used as a reference gene in human gastric cancer (Bednarz-Misa et al. 2021), human prostate cancer (Hsueh et al. 2012), and brain tumors (Vardon et al. 2017) for gene expression studies during L-arginine deprivation. *B2M, RPL13A, UBC,* and *YWAHAZ* were selected as they have been tested as potential reference genes for breast cancer cell lines in previous studies (Gorji-Bahri et al. 2021; Kiliç et al. 2014).

**Table 1:** Overview on reference genes used in this study

Gene abbreviation	Gene name	Molecular function	Assay ID
*ACTB*	Actin beta	Cytoskeletal component	Hs01060665_g1
*B2M*	Beta-2-microglobulin	Component of major histocompatibility complex	Hs00187842_m1
*GAPDH*	Glyceraldehyde-3-phosphate dehydrogenase	Glycolysis	Hs00266705_g1
*PPIA*	Peptidylprolyl isomerase A	Protein folding	Hs04194521_s1
*PUM1*	Pumilio RNA binding family member 1	RNA binding protein	Hs00472881_m1
*RPL13a*	Ribosomal protein L13a	Enabling mRNA binding activity	Hs04194366_g1
*SDHA*	Succinate dehydrogenase complex flavoprotein subunit A	Glycolysis	Hs00188166_m1
*TBP*	TATA-box binding protein	Initiation of transcription	Hs00427620_m1
*UBC*	Ubiquitin C	Stress regulation	Hs05002522_g1
*YWAHZ*	Tyrosine 3-monooxygenase/tryptophan 5-monooxygenase activation protein zeta	Mediation of signal transduction	Hs01122445_g1

*Assay ID* TaqMan assays from Thermo Fischer Scientific

### Quantitative real-time polymerase chain reaction (qRT-PCR)

Quantitative real-time polymerase chain reaction (qRT-PCR) was performed using TaqMan® real-time PCR assays labeled with 6-carboxyfluorescein (FAM-labeled; Applied Biosystems™, ThermoFisher Scientific, US). 5 µl TaqMan™ fast advanced Mastermix (Applied Biosystems™) and 0.5 µl of the 20 × TaqMan® assay were mixed in an Eppendorf tube and subsequently transferred to each well of the 96-well plate (MicroAmp® Optical Well Reaction Plate, Thermo Fisher Scientific). 4.5 µl of diluted cDNA (dilution of 1:45 ≙ 2.77 ng/µl) was used per reaction. Each reaction was performed in technical triplicates, and non-template controls were included in each assay. The Quantstudio 5 real-time PCR system (Thermo Fisher Scientific) was utilized to run the qRT-PCR reaction with the following cycling conditions: UNG incubation for 2 min at 50 °C; polymerase activation 10 min at 95 °C; followed by 40 cycles of denaturation at 95 °C for 15 s and annealing/extension at 60 °C for 1 min.

### Experimental conditions of L-arginine depletion

L-Arginine-free medium for the breast cancer cell lines MCF-7, BT-474, SK-BR-3, MDA-MB-468 and MDA-MB-231 was composed of 10.4 g/L powdered RPMI 1640 for stable isotope labeling with amino acids in cell culture (SILAC), 23.81 mM sodium bicarbonate, 0.38 mM L-leucine, and 0.26 mM L-lysine. 10% FBS, 100 units/ml penicillin, and 100 μg/ml streptomycin were added to the medium. L-Arginine depletion for the normal breast cell line MCF-12A was carried out in DMEM/F-12 for SILAC medium without L-arginine but supplemented with 0.4986 mM L-lysine, 5% HS, 100 units/ml penicillin, and 100 μg/ml streptomycin. Like in the L-arginine-containing medium, the additives 100 ng/ml cholera toxin, 20 ng/ml hEGF, 0.01 mg/ml insulin, and 500 ng/ml hydrocortisone were added immediately before use. Cell lines were seeded 24 h before incubation start, to allow cells to sink and attach. The incubation was started by replacing the L-arginine-rich medium with the incubation medium (L-arginine-rich or L-arginine-free). Cells were harvested after 24 h and 72 h at a confluence of 70–80%. Culture medium was changed every 24 h to ensure sufficient nutrient supply during the incubation period.

### Data analysis

Candidate reference gene stability was evaluated using four different algorithms. Mean quantification threshold cycle (Ct) values were used (1) to calculate the coefficient of variation (CV) and (2) for pairwise comparison of ΔCt values of candidate genes in each possible combination (Silver et al. 2006). (3) The Excel-based NormFinder estimates the overall gene expression variation for each potential gene and provides a stability value that is inversely proportional to its expression stability (Andersen et al. 2004). (4) RefFinder, a web-based tool (Xie et al. 2012), combines the four algorithms, NormFinder, geNorm (Vandesompele et al. 2002), BestKeeper (Pfaffl et al. 2004), and pairwise ΔCt to generate a comprehensive ranking based on the geometric mean (geomean) of their respective weights. The geometric mean is a measure of central tendency that calculates the nth root of the product of n values. These analyses were performed for each cell line individually and for all cell lines combined. To summarize the rankings of all algorithms, the rank of each candidate reference gene in each algorithm was converted into points. Specifically, rank 1 was assigned one point, rank 2 received two points, etc. Thus, a lower sum score across all algorithms indicated higher expression stability for the respective candidate reference gene.

In a separate approach, the complete set of analyses was repeated comparing cells cultured in L-arginine-rich or L-arginine-free medium.

## Results

### Gene expression profiles of selected candidate reference genes

**Fig. 1:**
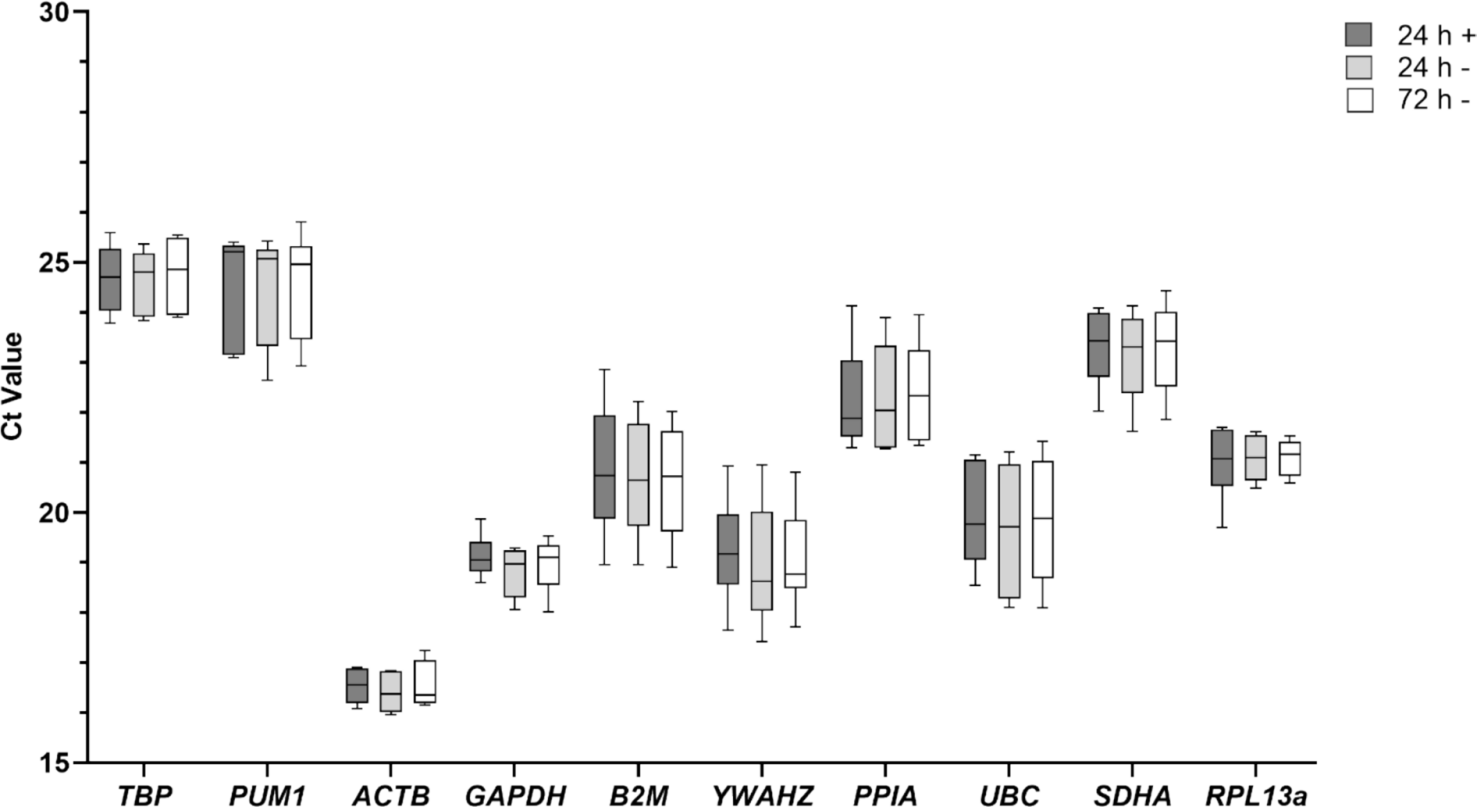
Absolute quantification cycle (Cq) values for 10 candidate reference genes. mRNA expression levels of 10 selected reference genes in 6 human breast cancer cell lines presented by Cq values. Cells were cultured in L-arginine-rich medium (+) and in L-arginine-free medium (−) for up to 72 h. The box represents the 25^th^ and 75^th^ percentile and the line in each box indicates the median. 10/90 percentile ranges are indicated by whiskers

Absolute Ct values were obtained to monitor expression levels of 10 putative reference genes ranging from the most abundantly (*ACTB*, mean Ct 16.49 ± 0.43) to the least abundantly expressed genes (*TBP*, Ct 24.69 ± 0.66; *PUM1*, mean Ct 24.52 ± 1.02). While some genes had a relatively wide variability of transcription across cell lines (e.g., *UBC* SD 1.1; *B2M* SD 1.1), other genes had a more uniform gene expression pattern (e.g., *ACTB* SD 0.43; *RPL13a* SD 0.53; *GAPDH* SD 0.51) (Fig. 1). The average CV for all genes under basal conditions was 3.81%, ranging from 2.33% for *GAPDH* to 5.82% for *B2M* (Table 2).

**Table 2:** Stability ranking of 10 candidate reference genes analyzed by four mathematical approaches across all cell lines under basal conditions

Rank	CV analysis		Pairwise ΔCt		NormFinder		RefFinder		Sum rank	
	Gene name	CV	Gene name	Mean SD	Gene name	Stability value	Gene name	Geomean	Gene name	Points
1	*GAPDH*	2.33%	*ACTB*	0.847	*ACTB*	0.015	*GAPDH*	1.410	*ACTB*	6
2	*ACTB*	2.39%	*GAPDH*	0.874	*GAPDH*	0.026	*ACTB*	1.570	*GAPDH*	6
3	*TBP*	2.62%	*RPL13A*	0.912	*TBP*	0.027	*TBP*	2.830	*TBP*	13
4	*SDHA*	3.12%	*TBP*	0.966	*RPL13a*	0.028	*RPL13a*	3.220	*RPL13a*	16
5	*RPL13a*	3.26%	*SDHA*	1.026	*SDHA*	0.031	*SDHA*	5.000	*SDHA*	19
6	*PUM1*	4.20%	*UBC*	1.046	*YWAHZ*	0.038	*PPIA*	6.480	*PPIA*	28
7	*PPIA*	4.42%	*YWAHZ*	1.095	*PPIA*	0.038	*UBC*	6.960	*UBC*	29
8	*UBC*	4.82%	*PPIA*	1.116	*UBC*	0.038	*YWAHZ*	7.670	*YWAHZ*	30
9	*YWAHZ*	5.14%	*PUM1*	1.127	*PUM1*	0.039	*PUM1*	8.970	*PUM1*	33
10	*B2M*	5.82%	*B2M*	1.158	*B2M*	0.050	*B2M*	9.740	*B2M*	40

Genes are ranked by increasing stability ratings for each method

*CV* coefficient of variation, *SD* standard deviation

### Identification of a suitable reference gene for comparison of gene expression in breast cancer cell lines under basal conditions

**Fig. 2:**
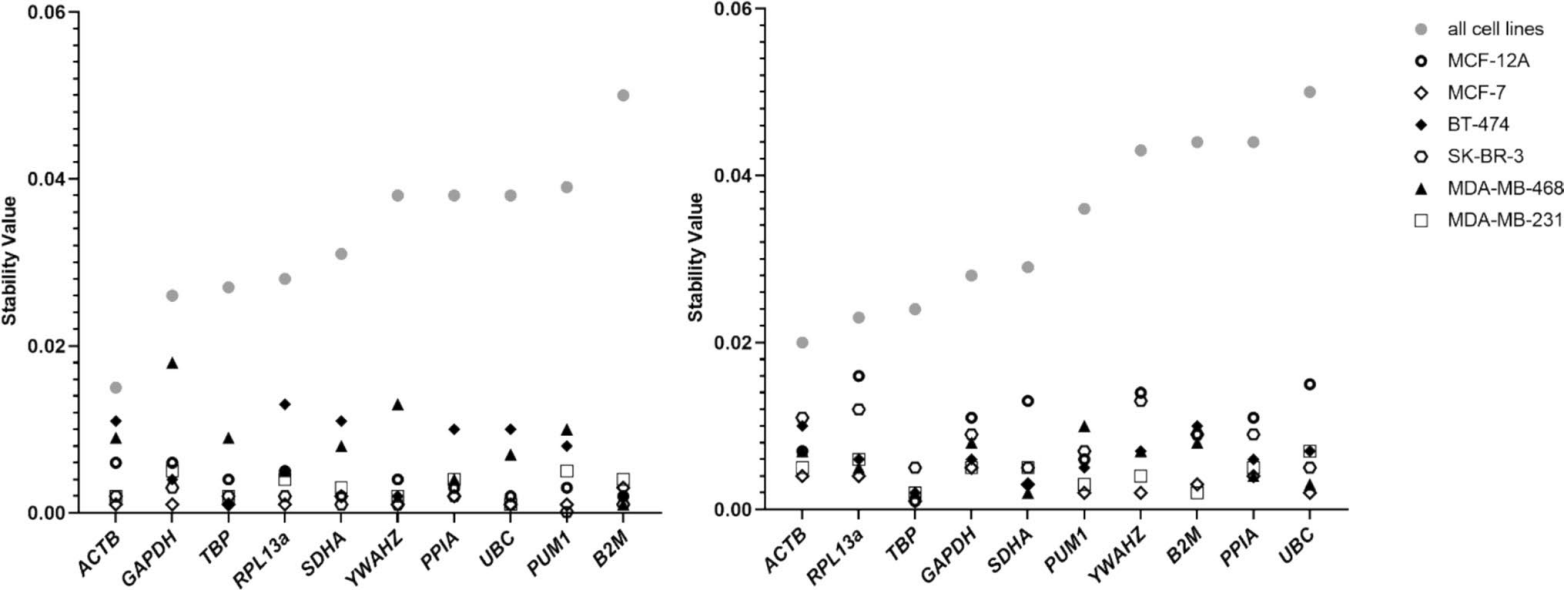
NormFinder results with stability values on the Y-axis and selected candidate genes on the X-axis for 6 different breast cell lines under basal conditions (left) and combined: basal conditions and L-arginine depletion (right). For each cell line, stability values were calculated separately. According to the expression stability, NormFinder ranks the set of possible reference genes with a cut-off value of 0.15. High expression stability of reference genes is reflected in low stability values. Please note that the order of the reference gene names on the x-axis differs between the left and right side

The pairwise ΔCt method (Silver et al. 2006) showed a low mean standard deviation (SD) for *ACTB* (0.847), whilst the highest expression variation was observed in *B2M* with an average SD of 1.158 (Table 2). Moreover, applying the NormFinder algorithm (Andersen et al. 2004), the most stable reference gene across all cell lines under basal conditions was *ACTB* (Fig. 2, left).

Table 2 shows the ranking results of all putative reference genes used in this study for all algorithms. While the ranking of reference genes varies across individual cell lines, *ACTB* and *GAPDH* were the two most stable reference genes when all cell lines were considered collectively. The sum scores of *GAPDH* and *ACTB* were identical (Table 2), so both genes shared the first rank under basal conditions. All tools ranked *PUM1* and *B2M* in one of the last ranks, with *B2M* having the lowest overall score. Evaluating the stability of the reference genes across the different cell lines, *UBC* showed highest stability values in MDA-MB-231 and MCF-7, while *B2M* is top ranked for MCF-12A and MDA-MB-468. SK-BR-3 has the most stable expression of *PUM1*, whereas BT-474 has *TBP* as the top ranked gene.

### Effects of L-arginine depletion on reference gene stability

The stability ranking was slightly different when comparing the combined data of basal conditions and L-arginine depletion with the results of the cells cultured under basal conditions. One of the most stable reference genes (*ACTB)* in the previous section also ranks among the top listed reference genes across the different algorithms in L-arginine-depleted cells. *RPL13a* was in the upper midfield under basal conditions and had the best stability value in the combined data set according to the CV analysis (CV 2.49%); it also was in second and third rank for the other algorithms. According to the NormFinder algorithm, different reference genes are top-ranked within the individual cell lines (Fig. 2, right). In MCF-12A and BT-474, *TBP* was top-ranked, whereas SK-BR-3 and MDA-MB-468 listed *SDHA* as top-ranked reference gene. The lowest stability values determined by the NormFinder algorithm were calculated for the genes *PUM1* in MCF-7 cells and *B2M* in MDA-MB-231 cells, respectively.

The total score of all algorithms combined was lowest for *ACTB*, followed by *RPL13a* and *GAPDH* (Table 3), making these genes the most stable ones in the combined data set in the breast cancer cell lines studied. *UBC* and *B2M* were ranked among the lowest in all analysis tools, with *B2M* having the lowest total score.

**Table 3:** Stability ranking of 10 candidate reference genes analyzed by four mathematical approaches across all cell lines under basal conditions and after L-arginine depletion combined

Rank	CV Analysis		Pairwise ΔCt		NormFinder		RefFinder		Sum Rank	
	Gene name	CV	Gene name	Mean SD	Gene name	Stability value	Gene name	Geomean	Gene name	Points
1	*RPL13a*	2.49%	*ACTB*	0.861	*ACTB*	0.018	*ACTB*	1.320	*ACTB*	5
2	*ACTB*	2.61%	*GAPDH*	0.869	*RPL13a*	0.024	*GAPDH*	2.060	*RPL13a*	9
3	*GAPDH*	2.67%	*RPL13A*	0.904	*TBP*	0.025	*RPL13a*	2.630	*GAPDH*	11
4	*TBP*	2.68%	*TBP*	0.949	*GAPDH*	0.028	*TBP*	2.830	*TBP*	15
5	*SDHA*	3.53%	*SDHA*	1.041	*SDHA*	0.029	*SDHA*	5.230	*SDHA*	20
6	*PUM1*	4.18%	*YWAHZ*	1.106	*PUM1*	0.037	*YWAHZ*	5.960	*PUM1*	27
7	*PPIA*	4.50%	*PUM1*	1.133	*YWAHZ*	0.042	*PPIA*	6.740	*YWAHZ*	28
8	*B2M*	5.36%	*UBC*	1.155	*PPIA*	0.042	*PUM1*	8.460	*PPIA*	32
9	*YWAHZ*	5.46%	*B2M*	1.168	*UBC*	0.046	*UBC*	9.000	*UBC*	36
10	*UBC*	5.55%	*PPIA*	1.180	*B2M*	0.046	*B2M*	9.460	*B2M*	37

Genes are ranked by increasing stability ratings for each method

*CV* coefficient of variation, *SD* standard deviation

## Discussion

This study shows that amongst ten genes that have previously been described as suitable reference genes in gene expression studies of various human tumors and / or in the comparison of malignant with normal breast tissue, a broad variation of expression stability of these putative reference genes can be observed across six different human breast cell lines. Whilst the results of different algorithms for assessing stability of expression varied, *ACTB* and *GAPDH* turned out to exhibit the best rank score across all four mathematical approaches. When metabolic restrictions like L-arginine depletion are applied, stability needs to be re-tested under these specific conditions. Here, *ACTB, RPLP13*, and *GAPDH* turned out to provide the best stability rankings.

Quantitative real-time polymerase chain reaction (qRT-PCR) is an accurate, rapid and highly accessible method for relative mRNA expression analysis in cancer tumorigenesis for development of new therapeutic interventions in breast cancer. To normalize gene expression, a reference gene is used that should have stable expression in the chosen experimental conditions (Vandesompele et al. 2002). A suitable reference gene should have a Ct value between 15 and 30 and an SD less than 1 to be considered stably expressed (Hruz et al. 2011; Van Acker et al. 2019). We selected 10 reference genes from the literature (Table 1), which were either reported to be the most stable genes in various human tissues from breast, human gastric cancer, human prostate cancer and brain tumors or are commonly used reference genes (Bednarz-Misa et al. 2021; De Kok et al. 2005; Gur-Dedeoglu et al. 2009; Hsueh et al. 2012; Kiliç et al. 2014; Lyng et al. 2008; Majidzadeh-A et al. 2011; McNeill et al. 2007; Morse et al. 2005; Salimi et al. 2012; Vardon et al. 2017). With Ct values ranging from 16 to 26, the reference genes used in this study were moderately abundant and showed high expression stability with low stability values (< 0.05), according to the NormFinder algorithm below the cut-off value of 0.15 (Andersen et al. 2004). However, using the pairwise ΔCt method, only four reference genes (*ACTB, GAPDH, RPL13a,* and *TBP*) would be considered stably expressed, as all other genes exceeded an SD of 1 (Table 3). The unique strategy of each algorithm to evaluate gene stability is likely to explain these differences in expression stability between algorithms.

Therefore, we used and compared four different algorithms to calculate the expression stabilities of candidates in order to identify an appropriate reference gene across six breast cell lines. The reference gene rankings of these methods were largely consistent, and *ACTB* appears to be the most stable reference gene across all cell lines studied, regardless of L-arginine depletion (Tables 2 and 3). These findings are supported by the literature, as *ACTB* was identified as one of the reference genes for human normal breast tissues and breast tumor (Gur-Dedeoglu et al. 2009; Majidzadeh-A et al. 2011; Morse et al. 2005).

However, ACTB was shown to not be a suitable reference gene in a study comprising three breast cancer cell lines (Gorji-Bahri et al. 2021) and in another study involving only MCF-7 cells (Jain et al. 2020). Together, these observations strongly support the view that even within one disease entity, stability of the reference gene used must be tested under the specific experimental conditions.

*GAPDH* is commonly selected in studies of vertebrate gene expression. However, *GAPDH* is known to be stimulated by various biological factors and is involved in biological processes (Chapman and Waldenström, 2015; Sirover 2005). Increased *GAPDH* expression has been reported in human cancers from prostate, lung and cervix (Kim et al. 1998; Rondinelli et al. 1997; Tokunaga et al. 1987), as well as in MCF-7 breast cancer cells treated with estradiol (Révillion et al. 2000). Consequently, *GAPDH* has not been proposed as a reference gene to study breast cancer (De Kok et al. 2005; Gur-Dedeoglu et al. 2009; McNeill et al. 2007; Révillion et al. 2000). Our results also showed that *GAPDH* was not one of the most stable candidates, in some cases being one of the lowest ranked genes when looking at cell lines and treatments individually. However*, GAPDH* is still widely used as a reference gene to normalize mRNA expression (Chapman snd Waldenström, 2015). As stated by Liu et al. (2015), caution should be exercised when using *GAPDH* as a reference gene for breast cancer studies and, ideally, appropriate reference gene analyses should be performed for a specific experimental setup in the respective tissues.

To the best of our knowledge, this study is the first to investigate the effect of L-arginine depletion on the stability of reference genes in breast cell lines. For evaluation of stabilities of candidate reference genes und basal conditions and after L-arginine depletion, we cultured six breast cell lines in culture medium with and without L-arginine. Data indicate that reference gene stability was influenced by cell line and treatment (Fig. 2). Although *ACTB* does not have the highest stability rankings in the individual cell lines, it has the most stable mRNA expression in all cell lines combined, both under basal conditions and combined basal conditions and L-arginine deprivation, and was therefore found to be a suitable reference gene for this experimental setup in the cell lines mentioned. Due to the heterogeneity of breast cancer, these results are not surprising and show once again how gene expression profiles of reference genes can vary between different cell lines and different experimental conditions. Consequently, ideal normalization of normal breast and breast cancer expression data requires analysis for the best possible reference gene among various known and formerly proposed reference genes in each sample group and experimental condition.

## Data Availability

All data supporting the findings of this study are available within the paper and its Supplementary Information.
